# *Roseomonas mucosa* infective endocarditis in patient with systemic lupus erythematosus: case report and review of literature

**DOI:** 10.1186/s12879-019-3774-0

**Published:** 2019-02-12

**Authors:** Shayuan Shao, Xin Guo, Penghao Guo, Yingpeng Cui, Yili Chen

**Affiliations:** 1 Department of Laboratory Medicine, The Seventh Affiliated Hospital of Sun Yat-sen University, Shenzhen, 518000 Guangdong China; 2 Department of Laboratory Medicine, Sun Yat-sen University, Guangzhou, 510080 Guangdong China; 3 Department of Laboratory Medicine, The First Affiliated Hospital of Sun Yat-sen University, Guangzhou, 510080 Guangdong China

**Keywords:** Bacteremia, Infective endocarditis, Systemic lupus erythematosus, *Roseomonas mucosa*, Case report

## Abstract

**Background:**

*Roseomonas mucosa*, as a Gram-negative coccobacilli, is an opportunistic pathogen that has rarely been reported in human infections. Here we describe a case of bacteremia in an infective endocarditis patient with systemic lupus erythematosus (SLE).

**Case presentations:**

A 44-year-old female patient with SLE suffered bacteremia caused by *Roseomonas mucosa* complicated with infective endocarditis (IE). The patient started on treatment with piperacillin-tazobactam and levofloxacin against *Roseomonas mucosa*, which was switched after 4 days to meropenem and amikacin for an additional 2 weeks. She had a favorable outcome with a 6-week course of intravenous antibiotic therapy.

**Discussion and conclusions:**

*Roseomonas mucosa* is rarely reported in IE patients; therefore, we report the case in order to improve our ability to identify this pathogen and expand the range of known bacterial causes of infective endocarditis.

## Background

The genus *Roseomonas* is a pink-pigmented, oxidative, mucosal Gram-negative coccobacilli, which is mostly isolated from environmental samples, such as water, soil, air and plants et al [1, 2]. *Roseomonas gilardii* (*R.gilardii*) and *Roseomonas cervicalis* were first described in 1993 [3]. *Roseomonas mucosa* (*R.mucosa*) was initially grouped with *R. gilardii*. However, due to the significant difference in genotype and phenotype, the isolated *R. mucosa* from a patient blood sample in 2003 was re-established as a distinct species [4]. It is reported that *R. mucosa* can be isolated from water [1], skin [5] and teeth root canal [6]. Although *R. mucosa* shows low human pathogenicity, it can lead to systemic infection with underlying diseases or immunocompromised patients, including patients with infectious spondylitis, peritonitis with HIV, and acute lymphoblastic leukemia [7–9]. Here we reported a case of bacteremia caused by *R. mucosa* in an infective endocarditis patient with systemic lupus erythematosus and additionally summarized a short review of infections with *R. mucosa*.

## Case presentation

A 44-year-old female patient was admitted with fatigue for 10 days, especially increased with shortness of breath after activities for the last 2 days. She announced that she had got cough, sore throat, with low-grade fever (without measuring) and pale face. About 2 months ago, she accepted the root canal therapy.

Upon physical examination, her heart rate was 96 beats/minute and regular. Body temperature was normal (36.7 °C). Laboratory examination showed the hemoglobin was 36 g/L, with a mean corpuscular volume of 78.9 fL, and the red blood cell was 1.42 × 10^12^/L, the reticulocyte was 2.52%. The color of the urine showed brown. Urinalysis showed that urobilinogen appeared positive (4+), with urine red blood cells 3cells/μL. The blood chemistry tests were shown as follows: lactic dehydrogenase (LDH) 594 U/L, serum total bilirubin (TBIL) 27.3 μmol/L, serum indirect bilirubin (IBIL) 20.45 μmol/L. According to the patient’s report, similar physical situation occurred 15 years ago, without further treatment. It was suspected that she was suffering autoimmune hemolytic anemia. Further examination of serum autoantibodies showed antinuclear antibodies (ANA) 188.35 IU/ml, anti-double-stranded DNA antibody (dsDNA) 186.42 IU/ml, anti-nucleosome antibodies (AnuA) 27.01 IU/ml, anti-SSA antibody (+), anticardiolipin antibodies (aCL) IgG (+), aCL-IgM (+), anti-β2-glycoprotein-I antibodies (a-β2-GPI) (+), Coombs test (4+). According to American College of Rheumatology (ACR) criteria, she was diagnosed as systemic lupus erythematosus (SLE). After red blood transfusion and hormonotherapy treatment with dexamethasone (10 mg qd), her clinical symptoms have improved.

**Fig. 1:**
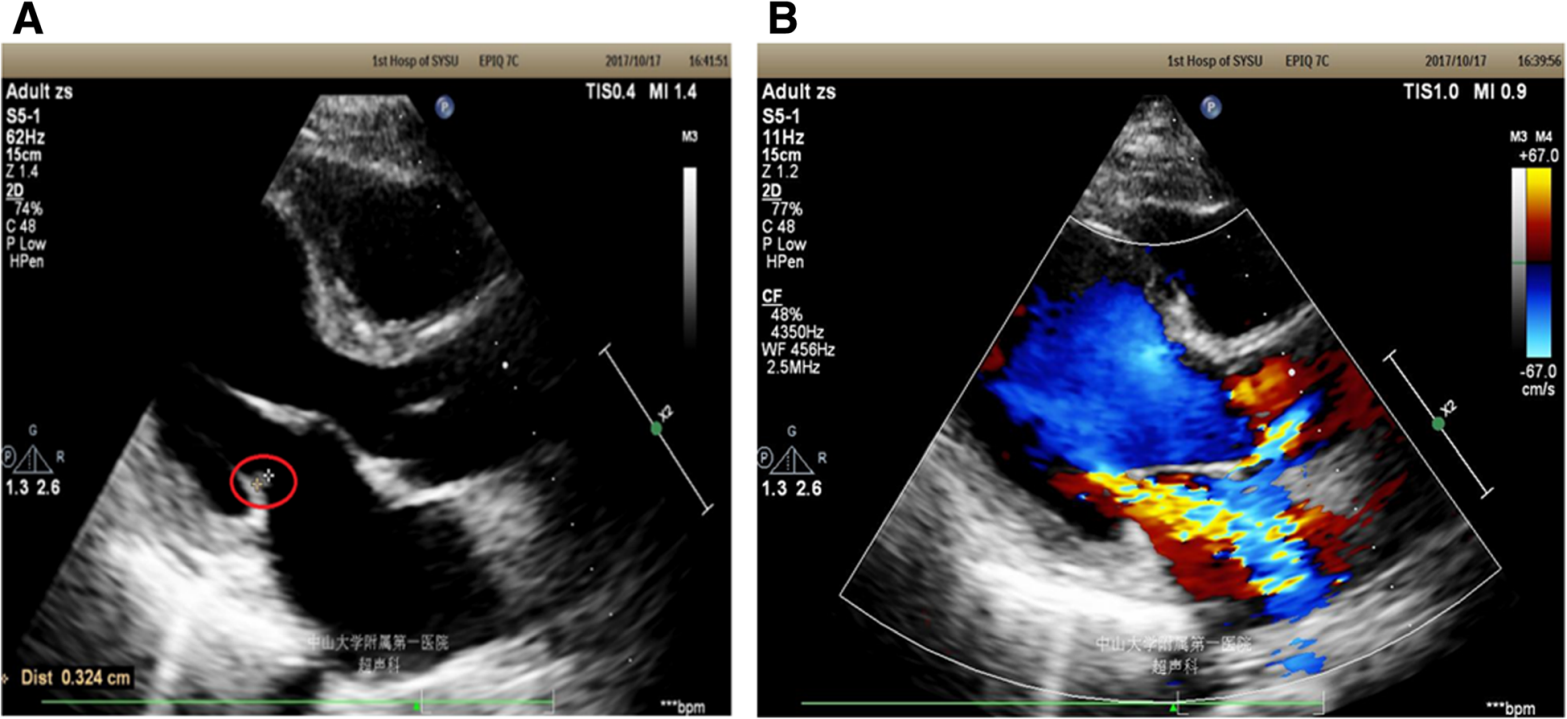
The echocardiograph images (TTE) indicated (**a**) existence of vegetation (as indicated by the red circle) and (**b**) acute severe mitral valve systole regurgitation

On the third day after admission, the transthoracic echocardiography (TTE) was performed as a routine examination. TTE results showed that the posterior leaflet of the mitral valve was thickened and incompetent. A small light band was observed on the mitral valve, which was fluttering along with the leaflet. A low echo mass was attached to the posterior leaf margin, which indicated vegetation existed. Its size was 3 mm × 3 mm. Acute severe mitral valve systole regurgitation was observed by Color Doppler Flow Imaging (CDFI), which indicated severe valvular inadequacy (Fig. 1).

**Fig. 2:**
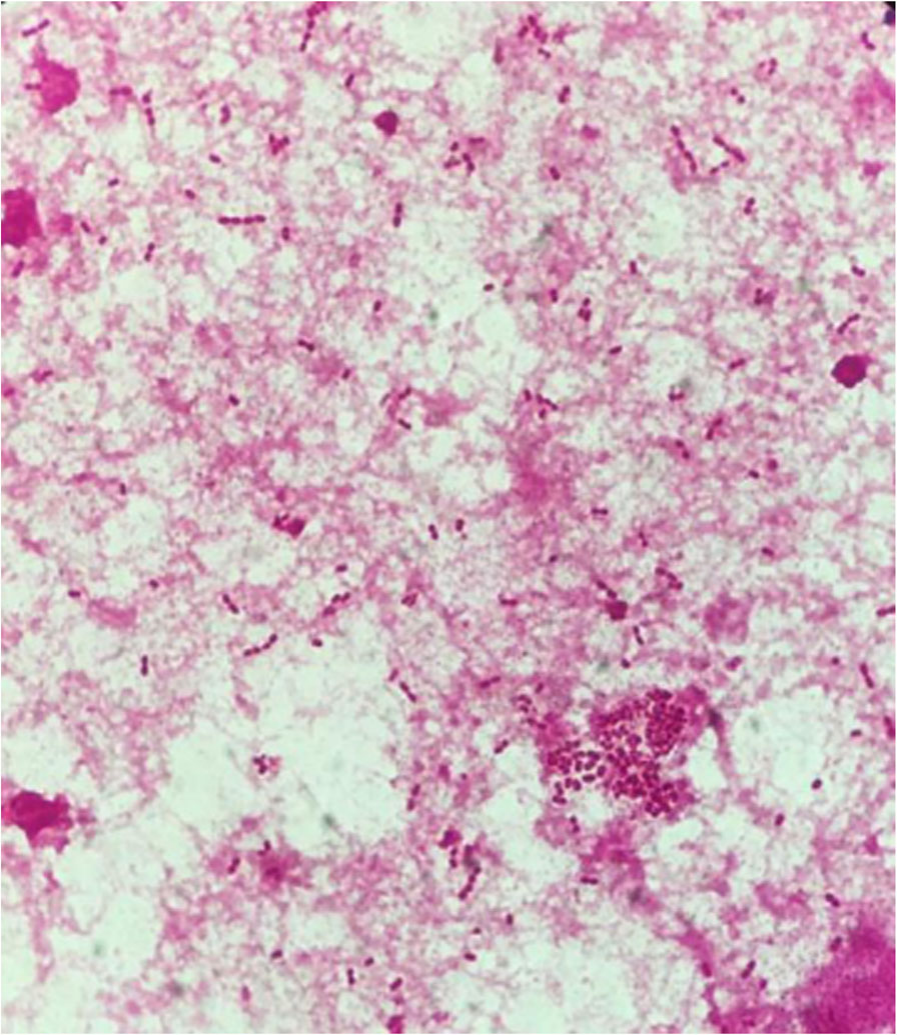
Gram staining of the blood culture isolated displayed Gram-negative coccobacilli, sometimes in short chains

Blood cultures were obtained then and empiric antibiotic treatment with vancomycin (1.0 g every 12 h) and piperacillin-tazobactam (4.5 g, iv drip q8h) were started. All the 6 sets of blood cultures were positive after 2 days and detected as Gram-negative coccobacilli (Fig. 2) The organism was subcultured and incubated on blood agar at 35 °C with 5% CO_2_. In the meantime, the empiric antibiotic treatment for the patient was switched to piperacillin-tazobactam (4.5 g, ivdrip q8h) and levofloxacin (0.5 g, ivdrip qd). The organism appeared slightly pink, mucoid colonies after 48 h incubation. It was identified as *R.mucosa* by matrix-assisted laser desorption ionization time-of-flight mass spectrometry (MALDI-TOF MS) (bioMerieux, Durham, NC). However, the organism was identified as *R.gilardii* by the Vitek2 system (bioMerieux, Durham, NC, USA). To confirm the identity of the isolate, a fragment of the 16S rRNA gene was amplified by PCR using primer sets 16S-forward (5′AGAGTTTGATCCTGGCTCAG 3′) and 16S-reverse (5′GGTTACCTTGTTACGACTT 3′), and the resultant polymerase chain reaction product was sequenced. The best match returned was the *R.mucosa,* ATCC BAA-692 type strain, with 99.6% identity.

**Fig. 3:**
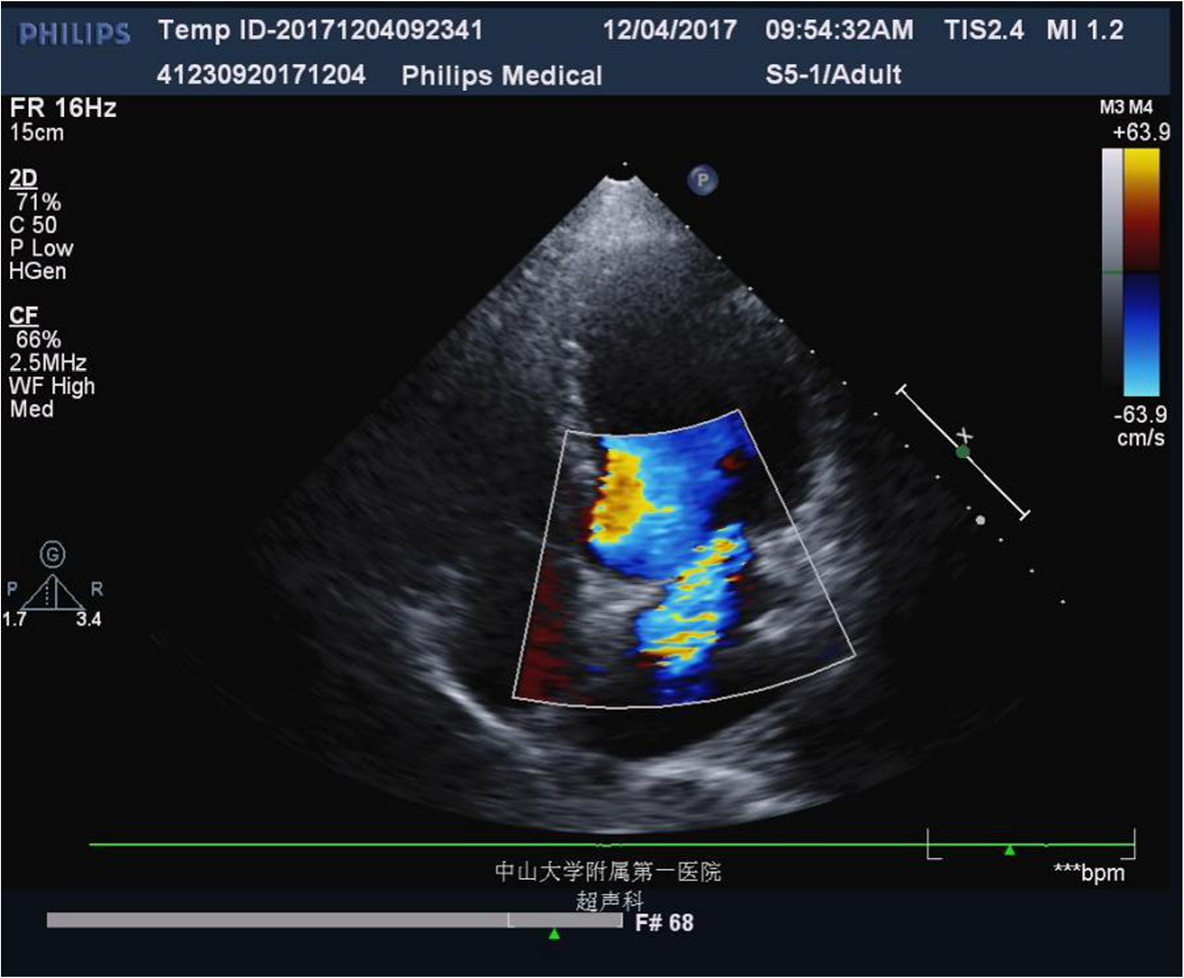
The control echocardiograph images (TTE) indicated moderate mitral valve systole regurgitation

Antimicrobial susceptibility testing of the *R.mucosa* strain was determined by the Kirby-Bauer disk diffusion method, using the breakpoints recommended by Clinical and Laboratory Standards Institute (CLSI-M100) for nonfermentative Gram-negative bacteria. The isolate exhibited large inhibition zone (millimeter) for most of antimicrobials tested: amikacin 42 mm, ciprofloxacin 42 mm, levofloxacin 27 mm, imipenem 38 mm, meropenem 42 mm, and piperacillin-tazobactam 6 mm. Therefore, according to the antibiotics susceptibility test result, the treatment was switched to meropenem (1 g, ivdrip q12h) and amikacin (400 mg, ivdrip qd). After antibiotic treatment, the control echocardiography showed that moderate mitral valve systole regurgitation was observed by CDFI, which was much better than before (Fig. 3). The following blood cultures, the sputum culture and urine culture were all negative and the C-reactive protein (CRP), the procalcitonin (PCT), the white blood cell counts and the neutrophil counts were all normal. After treatment, the hemoglobin has raised to 81 g/L. The results of serum autoantibodies, including ANA 130.04 IU/ml, dsDNA121.18 IU/ml, AnuA 19.45 IU/ml, anti-SSA antibody (±), were improved. The patient discharged and kept on accepting the treatment with meropenem and amikacin in community hospital for another 6 weeks until the clinical symptoms of the SLE were controlled. The patient is still preparing for a cardiac surgery which has been advised by the doctor.

## Discussion and conclusions

Infective endocarditis (IE) is a rare, life-threatening disease. However, the diagnosis of IE usually requires a combination of clinical microbiological and echocardiography results [10]. Although SLE itself could lead to the formation of vegetations of valve, which is a type of non-infective endocarditis and named as Libman-Sacks endocarditis [11]. According to the modified Duke Criteria, these were the certain evidences supporting the diagnosis of infective endocarditis in this case, including the positive of all the 6 sets of blood cultures, the existence of the vegetation on mitral valve by TTE, the presence of acute severe mitral valve systole regurgitation by CDFI examination, and the improved situation of mitral valve systole regurgitation after antibiotic treatment. Additionally, Libman-Sacks endocarditis vegetations are not generally associated with valvular destruction [12]. It is believed that valve tissue culture was important to distinguish IE from Libman-Sacks endocarditis in this case.

Almost 80% of IE cases were caused by Gram-positive pathogenic bacteria, such as viridans group *Streptococci* (VGS), *Streptococci* and *Staphylococci* [10, 13]. Infections involving Gram-negative and fungal pathogens in IE are rarely reported. Few studies showed that the incidence of non-HEACK (*Haemophilus* species, *Aggregatibacter* species, *Cardiobacterium hominis*, *Eikenella corrodens* and *Kingella* species) Gram-negative bacilli IE was increasing from 1.8% [14] to 3.9% [15]. In this case, IE caused by this slow-growth non-fermentative Gram-negative bacterium was rarely studied. *R. mucoca* was a bacterial genus of pink-pigmented, oxidative, Gram-negative coccobacilli, which grows well on blood agar and chocolate agar, but not on McConkey agar [4, 7–9]. In our case, the pathogen organism was misidentified as *R.gilardii* by the Vitek 2 system. However, according to the results of MALDI-TOF MS and 16S rRNA gene sequence [7], it was further identified as *R.mucoca*. Similar to our observation, there were also other researchers reported the misidentification with Vitek 2 system [16].

In general, *R.mucoca* was resistant to β-lactam antibiotics, such as piperacillin-tazobactam, ampicillin, extended spectrum cephalosporins (cefrazidime, cefepime) and colistin, while it was full susceptibility to aminoglycosides (amikacin, gentamicin) and fluoroquinolones (levofloxacin, ciprofloxacin), and usually susceptible to carbapenems (imipenem, meropenem) [2, 7–9]. Consistent to our results, the previous case series reported that 100% of *R.mucoca* isolated was resistant to piperacillin-tazobactam [17]. According to the study of a contemporary multicenter cohort in Italy, approximately 73% of non-HEACK Gram-negative bacilli IE patients were treated with penicillin or cephalosporin (penicillin–penicillinase inhibitor or a third-generation cephalosporin), variably combined with carbapenem and aminoglycoside or fluoroquinolone [15]. Since these β-lactam antibiotics usually had no effects on *R.mucoca* infections, it is believed that the management of IE caused by *R.mucoca* would be better to be treated with carbapenems combined with an aminoglycoside or a fluoroquinolone.

Usually, *Roseomonas spp.* were considered as a kind of opportunistic pathogen because of their low pathogenic potential in human [7]. Most *Roseomonas* infections have been reported in patients with immunocompromised host with underlying illnesses, including immunosuppressive of steroid therapy, chronic renal disease, liver cirrhosis, diabetes, rheumatoid arthritis, malignancy, organ transplantation and AIDS [8, 18], but cases have also been reported in the immunocompetent patient [7] (Table 1). The organism has been recovered from multiple clinical sources, including bloodstream, peritoneal dialysis fluid, bone and wounds [8]. Using 16S rRNA gene sequence revealed that *R.mucoca* was the most prevalent species among *Roseomonas* spp. in clinical samples, that can cause bacteremia, peritonitis, abscess formation, and infectious spondylitis [7, 16, 19].

**Table 1:** Summary of reported cases of *Roseomonas spp.* infection

Reference	Age (yr)/Sex	Infection	Underlying disease	Treatment	Outcome
Tsai S et al., 2012 [19]	48/M	Peritonitis	IgA nephropathy-related end-stage renal disease	Day1–6:cephalosporin Day7–18:ciprofloxacin Day19–72:cefazolin and gentamicin	Recovered
Boyd MA et al., 2012 [8]	19/M	Peritonitis	Chronic renal failure, AIDS	Cycling peritoneal dialysis (CCPD)	Failure.
Ece G et al., 2013 [16]	30/M	Abscess formation	Cranial fracture	Remove the cranioplasty	Recovered
Al-Anazi et al., 2013 [20]	41/F	Bacteremia	T-lymphoblastic lymphoma	Day1–4:Piperacillin-tazobactam 4.5 g IVq 8 h Day4–14:imipenem and amikacin Ciprofloxacin	Recovered
Kaore NM et al., 2014 [21]	37/M	(Community acquired secondary bacterial infections)	Pulmonary tuberculosis	Amikacin and Cefoperazone/Sulbactam	Recovered
Michon AL et al., 2014 [17]	3/M	Bacteremia	Acute lymphoblastic leukemia	Day1–4:piperacillin-tazobactam Day7–14:cefixime	Recovered
Kim KY et al., 2015 [7]	74/M	Infectious spondylitis with bacteremia	Compression fractures of the thoracic and lumbar spine	Day4–7:intravenous cefazolin Day8–14:ceftazidime	His back pain improved
Kim KY et al., 2016 [2]	84/F	Bacteremia	Cholecystitis and chol-angitis	Day4–6:cefotaxime and metroni-dazole combination	Recovered (no signs of infection)
Kim KY et al., 2016 [2]	17/M	Bacteremia	Acute myeloid leukemia	Vancomycin and carbapenem	No exhibit significant clinical signs
This present case	44/F	Infective endocarditis (IE)	Systemic lupus erythematosus (SLE)	Day4–7:vancomycin and piperacillin-tazobactam Day8–11:piperacillin-tazobactam and levofloxacin Day12–25:meropenem and amikacin	Recovered

In the present case, bacteremia was caused by *R.mucoca* in an infective endocarditis patient with systemic lupus erythematosus. We suspect that the source of the infection by *R.mucosa.* in this case may be due to the root canal therapy performed 2 months ago. The opportunistic pathogen entered the immunocompromised body through a small cut in the mouth and led to the bacteremia and formed the cardiac valve excrescent. A similar case was reported by Nina Diesendorf et al. which described a *R.mucosa* isolated from an infected tooth and suspected that infected root canal might serve as an entrance pathway for bloodstream infections by this opportunistic pathogen [6]. Therefore, immunocompromised patients with IE should be concerned with detailed discussion for their periodontal disease history. Additionally, it will be very important to identify pathogens in the early stage for useful treatment.

To our knowledge, this is the first report of *R.mucoca* infective endocarditis. Our report, a severe infectious complication in a patient with SLE, expands the scope of known bacterial etiology of infective endocarditis. MALDI-TOF MS and molecular analysis will provide more accurate identification of these organisms, since traditional biochemical identification equipment might misidentify them at the species level.

## Abbreviations

CDFI: Color Doppler Flow Imaging
IE: Infective endocarditis
MALDI-TOF MS: Matrix-Assisted Laser Desorption/ Ionization Time of Flight Mass Spectrometry
*R.gilardii*: *Roseomonas gilardii*
*R.mucoca*: *Roseomonas mucosa*
SLE: Systemic lupus erythematosus
TTE: Transthoracic echocardiography
